# Genetic variation in *Austrostrongylus thylogale* Johnston & Mawson, 1940 (Nematoda: Trichostrongylida) from the tammar wallaby, *Notamacropus eugenii* (Gray), and the quokka, *Setonix brachyurus* (Quoy & Gaimard) (Marsupialia: Macropodidae) in Australia

**DOI:** 10.1186/s13071-020-4007-5

**Published:** 2020-03-14

**Authors:** Tanapan Sukee, Tony Huynh, Ian Beveridge, Abdul Jabbar

**Affiliations:** Department of Veterinary Biosciences, Melbourne Veterinary School, The University of Melbourne, Werribee, VIC 3030 Australia

**Keywords:** *Austrostrongylus thylogale*, Tammar wallaby, Quokka, Internal transcribed spacers, Cryptic species

## Abstract

**Background:**

Australian marsupials harbour a diverse array of helminth parasites. Despite current attempts to assess the extent of this diversity in macropodid hosts, it has been suggested that unique parasite fauna of Australian wildlife is difficult to document comprehensively due to the common occurrence of cryptic species. This paper assessed genetic variation within *Austrostrongylus thylogale* Johnston & Mawson, 1940 from the tammar wallaby, *Notamacropus eugenii* (Gray), and the quokka, *Setonix brachyurus* (Quoy & Gaimard), from different localities using the molecular characterisation of the internal transcribed spacers (ITS) within the nuclear ribosomal DNA.

**Methods:**

Thirty-seven specimens of *A. thylogale* collected from *N. eugenii* (from Parndana, Kangaroo Island, South Australia, and Perup, Western Australia) and *S. brachyurus* (from Wellington Dam, Western Australia) were characterised using a molecular-phylogenetic approach utilising the first (ITS1) and second (ITS2) internal transcribed spacers.

**Results:**

Genetic variation was detected in both ITS1 and ITS2 between specimens of *A. thylogale* from *N. eugenii* and *S. brachyurus*; however, no variation was detected between specimens collected from *N. eugenii* from Parndana, South Australia, and Perup, Western Australia. Furthermore, the phylogenetic analyses of ITS sequences showed two clades of *A. thylogale* originating from two hosts, *N. eugenii* and *S. brachyurus*, suggesting the presence of cryptic species.

**Conclusions:**

This study provides evidence of genetic variation within *A. thylogale* based on collections from two different host species. Morphological studies are required to fully confirm the presence of a new species or cryptic species. Further molecular studies using a larger number of specimens are warranted to explore the genetic variation between *A. thylogale* from different geographical localities.
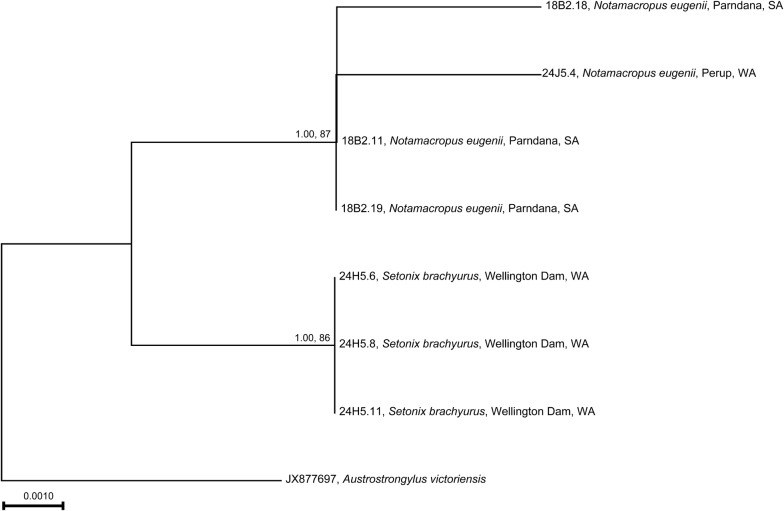

## Background

The Australian continent is biologically diverse, and native animals such as kangaroos and wallabies (family Macropodidae) harbour a diverse range of unique nematodes [1]. The abundance and extensive range of parasites has been attributed to the host-specific nature of the helminths found in macropodids and their herbivorous grazing habits [2, 3]. Given the large numbers of nematodes, host specificity, and variable size and morphology of worms, uncommon species may often be overlooked [3–5].

While macropodid nematodes are diverse, they are well known for having simple body plans and morphological uniformity, which can make it difficult to distinguish between species based on morphology alone [6]. As a result, molecular methods such as DNA-based techniques have been developed to enable the definition and identification of genetic markers for the characterisation of various species [7]. A commonly used genetic method in the identification of such nematodes is the sequencing of the first and second internal transcribed spacers (ITS1 and ITS2, respectively) of the nuclear ribosomal DNA, which are known to be reliable genetic markers due to the homology of these sequences between species [7, 8]. This molecular technique has been used in many studies to distinguish between morphologically similar species as well as cryptic (i.e. genetically distinct but morphologically similar) species [7, 9, 10].

The trichostrongylid genus *Austrostrongylus* Chandler, 1924 contains 13 recognised species, which occur in the duodenum of various macropodid hosts with an aberrant species in the marsupial mole (*Notoryctes typhlops* (Stirling)) [11–13]. *Austrostrongylus thylogale* Johnston & Mawson, 1940, was first described from tammar wallabies (*Notamacropus eugenii* (Gray)) on Kangaroo Island, South Australia [14] and was subsequently reported from quokkas (*Setonix brachyurus* (Quoy & Gaimard)) on Rottnest Island, Western Australia by Inglis [15]. *Notamacropus eugenii* occurs on Kangaroo Island, South Australia and, in a highly disjunct distribution, in the south-west of Western Australia including islands such as Garden Island. The population of *S. brachyurus* is distributed in the south-west of Western Australia, as well as on two offshore islands, Rottnest Island and Bald Island [16]. The distribution of *N. eugenii* is therefore sympatric with *S. brachyurus* in the south-west of Western Australia. A recent study analysing the ITS sequences of mainland and island populations of the strongylid nematode *Labiosimplex australis* Kung, 1948 suggested that genetic variation within this species was possibly due to geographical isolation on islands or between geographically distant locations [17]. Therefore, we hypothesised that similar genetic differences might also be present in populations of *A. thylogale* as it has a disjunct geographical distribution and occurs in different host species [6]. This paper aimed to assess the genetic variation in *A. thylogale* collected from different localities and host species by characterising the ITS region of the nematode.

## Methods

### Collection of specimens

Adult specimens of *A. thylogale* (*n* = 37) were sourced from the frozen parasite collection at the School of Veterinary Science, The University of Melbourne. These specimens had been collected opportunistically from culled or road-killed hosts from various localities in Australia (Table 1). The nematodes were frozen in liquid nitrogen and stored at − 80 °C until use. Upon thawing, the anterior and posterior ends were excised, placed in lactophenol and stored for later morphological analysis. Mid-sections were used for genomic DNA extraction. Morphological vouchers for the specimens sequenced were deposited in the South Australian Museum, Adelaide: from *N. eugenii*, Perup River, Western Australia (24J5.4) AHC 48324; Parndana, Kangaroo Island, South Australia (18B2.2, 2.9, 2.11, 2.18, 2.19) AHC 48326; from *S. brachyurus*, Wellington Dam, Western Australia (24H5.2, 5.5, 5.6, 5.8) AHC 48325.

**Table 1:** *Austrostrongylus thylogale* samples used for molecular analysis with descriptions of host species, *Notamacropus eugenii* and *Setonix brachyurus*, localities in South Australia (SA) and Western Australia (WA) and GenBank accession numbers

Sample	Host species	Locality	GenBank ID	
			ITS1	ITS2
18B2.9	*N. eugenii*	Parndana, SA	–	–
18B2.11	*N. eugenii*	Parndana, SA	MT022443	MT022450
18B2.14	*N. eugenii*	Parndana, SA	MT022444	–
18B2.18	*N. eugenii*	Parndana, SA	MT022445	MT022451
18B2.19	*N. eugenii*	Parndana, SA	–	MT022452
24J5.4	*N. eugenii*	Perup, WA	MT022446	MT022453
24J5.5	*N. eugenii*	Perup, WA	–	–
24J5.6	*N. eugenii*	Perup, WA	MT022447	–
24J5.8	*N. eugenii*	Perup, WA	MT022448	–
24H5.2	*S. brachyurus*	Wellington Dam, WA	–	MT022454
24H5.6	*S. brachyurus*	Wellington Dam, WA	–	–
24H5.8	*S. brachyurus*	Wellington Dam, WA	–	–
24H5.10	*S. brachyurus*	Wellington Dam, WA	–	–
24H5.11	*S. brachyurus*	Wellington Dam, WA	MT022449	–

Note: – indicates identical sequences

### Molecular characterisation

Total genomic DNA (gDNA) was isolated from individual worms using the Wizard SV Genomic DNA Purification kit (Promega, Madison, WI, USA). Both the first (ITS1) and second (ITS2) sequences were amplified by one PCR reaction using primers NC16 (5′-AGT TCA ATC GCA ATG GCT T-3′) and NC2 (5′-TTA GTT TCT TTT CCT CCG CT-3′) [7]. PCRs were conducted in 50 μl volumes containing 2 µl of DNA template, 10 mM Tris-HCl (pH 8.4), 50 mM KCl (Promega), 3.5 mM MgCl_2_, 250 μM of each deoxynucleotide triphosphate (dNTP), 100 pmol of each primer, and 1 U of GoTaq polymerase (Promega). The PCR conditions used were: 5 min at 94 °C, then 35 cycles of 30 s at 94 °C, 20 s at 55 °C, and 20 s at 72 °C, followed by 5 min at 72 °C. Negative (no DNA template) and positive controls (*Trichostrongylus colubriformis* (Giles, 1892) gDNA) were included in the PCR analyses. An aliquot (5 μl) of each amplicon was subjected to agarose gel (1.5%) electrophoresis to visualise PCR amplicons.

Amplicons were purified using shrimp alkaline phosphate and exonuclease I [18] prior to automated Sanger DNA sequencing at Macrogen Incorporation, South Korea, using the same primers used in the PCR.

### Sequence and phylogenetic analyses

The quality of each forward and reverse sequence was assessed, and each consensus sequence assembled using Geneious Prime 2019.0.4 (www.geneious.com). Polymorphic sites were designated using the International Union of Pure and Applied Chemistry codes. All nucleotide sequences obtained were deposited in the GenBank (Table 1). Nucleotide sequences were aligned using MUSCLE v.3.8.31 [19] within MEGA-X using default settings [20]. Pairwise comparisons of aligned sequences were performed to calculate nucleotide differences using BioEdit [21].

Unique ITS1, ITS2 and concatenated ITS (designated as ITS+) sequences collected in this study were aligned with respective reference sequences using MUSCLE in MEGA using default settings and were trimmed to uniform lengths of 356 bp (ITS1), 240 bp (ITS2) and 596 (ITS+) bp. The evolutionary models for each DNA sequence dataset [ITS1 and ITS+: Hasegawa-Kishino-Yano (HKY); ITS2 Tamura-Nei (TrN)] were determined using the Akaike and the Bayesian information criteria (AIC and BIC) tests in jModelTest v.3.7 [22]. Neighbour-joining (NJ) trees were constructed using MEGA, and Bayesian Inference (BI) trees were built using MrBayes [23]. The NJ trees were constructed with 10,000 bootstrap replicates using the Tamura-Nei distance method. Each BI analysis was run for 20,000,000 generations to calculate posterior probabilities (pp), with two runs, with every 200th tree saved. Sequences from *Austrostrongylus victoriensis* Cassone, 1983 were included as the outgroup. Tree topology was checked for consensus between NJ and BI analyses using the software Figtree v1.4.4 (http://tree.bio.ed.ac.uk/software/figtree/).

## Results and discussion

Following the PCR amplification of the ITS region, 14 amplicons were selected for DNA sequencing. Six and five unique sequences of ITS1 (length: 399 bp) and ITS2 (length: 240 bp) were identified, respectively. The G+C content of ITS1 sequences of *A. thylogale* was higher in specimens collected from *S. brachyurus* (42.6%) than those obtained from *N. eugenii* (41.5–41.6%), with no difference based on location. However, the G+C content of ITS2 sequences was higher in specimens of *A. thylogale* collected from *N. eugenii* (Parndana: 43.8–45.0%; Perup: 44.2%) than those collected from *S. brachyurus* (44.6%).

The ITS1 sequences of *A. thylogale* from the Parndana population of *N. eugenii* had fewer nucleotide polymorphisms [two nucleotide positions: 353 (W) and 397 (R)] than those from *S. brachyurus* [four nucleotide positions: 144 (S) 208 (K), 244 (M) and 274 (R)]. Furthermore, the nucleotide variability between *A. thylogale* from *N. eugenii* and *S. brachyurus* was linked to three transitions (T ↔ C or A ↔ G) (Table 2; see Additional file 1: Figure S1). Based on within-host species pairwise comparisons, higher nucleotide variation was observed in specimens of *A. thylogale* collected from *S. brachyurus* (0.3–2.3%) than those collected from *N. eugenii* (0.3–1.8%).

**Table 2:** Nucleotide positions showing the variation within the first internal transcribed spacer sequences of *A. thylogale* from two hosts

Host	Specimen ID	Alignment positions								
		54	144	208	244	274	288	318	353	397
*Notamacropus eugenii*	18B2.11	T	G	G	A	G	A	A	A	G
	18B2.14	T	G	G	A	G	A	A	**W**	**R**
	18B2.18	T	G	G	A	G	A	A	A	**R**
*Setonix brachyurus*	24H5.6	**C**	G	G	A	G	**G**	**G**	A	G
	24H5.8	**C**	**S**	G	**M**	**R**	**G**	**G**	A	G
	24H5.11	**C**	G	**K**	A	G	**G**	**G**	A	G

*Abbreviations*: A, adenine; T, thymine; G, guanine; C, cytosine, S, C or G; M, A or C; K, T or G; R. A or G; W, A or T

Nucleotide differences of 0.5–1.7% from pairwise comparisons of the ITS2 sequences of *A. thylogale* from the Parndana population of *N. eugenii*, were mainly attributed to nucleotide polymorphisms (two nucleotide positions: 2 (Y) and 148 (R)), two transitions (T ↔ C) and two transversions (T ↔ A or A ↔ C) (Table 3; see Additional file 1: Figure S1), whereas sequences from *S. brachyurus* were identical. The nucleotide variability between specimens of *A. thylogale* from *N. eugenii* and *S. brachyurus* was linked to one transition (T ↔ C) (Table 3; see Additional file 1: Figure S1).

**Table 3:** Nucleotide positions showing the variation within the second internal transcribed spacer sequences of *A. thylogale* from two hosts

Host	Specimen ID	Alignment positions						
		22	58	118	148	151	225	226
*Notamacropus eugenii*	18B2.11	**Y**	T	C	**R**	T	C	T
	18B2.18	T	T	**T**	G	**A**	C	T
	18B2.19	T	T	C	G	T	C	T
	25J5.4	T	T	C	G	T	**A**	**C**
*Setonix brachyurus*	24H5.2	T	**C**	C	G	T	C	T

*Abbreviations*: A, adenine; T, thymine; G, guanine; C, cytosine; R, A or G; Y, C or T

**Fig. 1:**
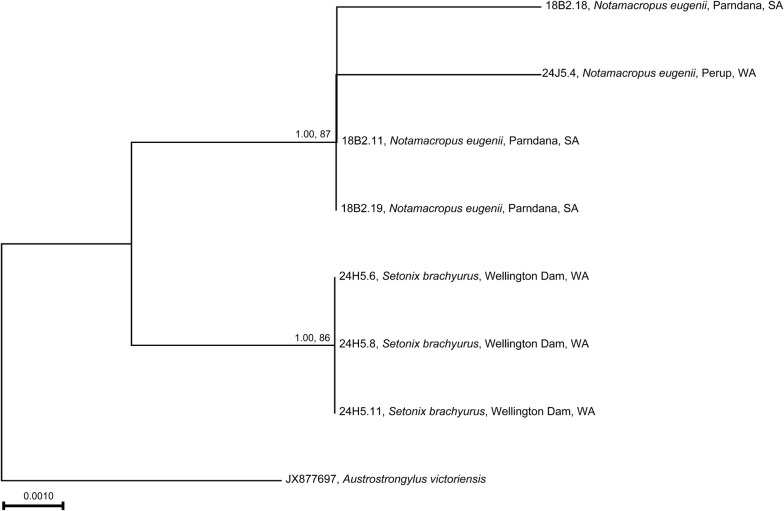
Phylogenetic analysis of the ITS+ rDNA sequences of *Austrostrongylus thylogale* from quokkas and tammar wallabies from different geographical locations. The sequence data were analysed using the Bayesian Inference (BI) and Neighbour-Joining (NJ) methods. Nodal support is given as a posterior probability and bootstrap value for BI and NJ, respectively. Unique sequences are presented with the voucher number, host and locality. *Austrostrongylus victoriensis* (JX877697) was used as the outgroup. The scale-bar indicates the number of inferred substitutions per nucleotide site. *Abbreviations*: SA, South Australia, WA, Western Australia

Phylogenetic analyses of separate ITS sequences using NJ and BI methods revealed that the topology of both trees generated (separately) for each dataset (ITS1, ITS2 and ITS+ sequences) were similar using BI and NJ (data not shown); hence, only the NJ tree of the ITS+ sequences is presented here (Fig. 1). The ITS sequences of *A. thylogale* from *N. eugenii* originating from Parndana (South Australia) and Perup (Western Australia) grouped together with strong statistical support (posterior probability of BI = 1.00, bootstrap value of NJ = 87%) whereas those from *S. brachyurus* formed the separate clade, also with strong statistical support (1.00, 86%) (Fig. 1).

To our knowledge, this is the first report of the genetic characterisation of the ITS+ DNA of *A. thylogale* and findings of this study support our hypothesis that specimens of *A. thylogale* collected from two different hosts i.e. *N. eugenii* and *S. brachyurus* are genetically distinct, suggesting the existence of cryptic species. Similar findings have been reported for a number of other macropodid nematodes [17, 24–28].

We found little variation in the ITS sequences of *A. thylogale* specimens collected from *N. eugenii* from two distant geographical locations (Parndana, South Australia, and Perup, Western Australia); however, there were four (3 in ITS1; 1 in ITS2) fixed nucleotide differences between specimens collected from *N. eugenii* and *S. brachyurus* (see Tables 2 and 3). Furthermore, the overall pairwise nucleotide differences and G+C content were also different between the specimens of *A. thylogale* originating from the two different host species. The ITS loci are known to be reliable genetic markers for the characterisation of strongylid nematodes as both ITS1 and ITS2 sequences can be quite conservative and only one or more fixed differences can assist in assessing interspecific differences [7, 29]. Therefore, the findings of this study suggest that *A. thylogale* may represent more than one species. However, this hypothesis needs to be tested using the morphological and molecular examination of a large number of specimens. Preliminary morphological examination of specimens from the two host species revealed no obvious differences.

Phylogenetic analysis of the ITS1, ITS2 and ITS+ sequences consistently revealed that *A. thylogale* from *N. eugenii* and *S. brachyurus* formed two separate clades (Fig. 1), and individual sequences from the same host species clustered together, suggesting this grouping is more strongly associated with the host rather than the locality.

Species of *Austrostrongylus* occur predominantly in the related wallaby genera *Notamacropus* Dawson & Flannery, *Dorcopsis* Schlegel & Müller and *Wallabia* Trouessart [1] although transmission occasionally occurs to other sympatric macropodid hosts although at a lower prevalence and intensity [30]. In this instance, it appears to be a host switch from *N. eugenii* to S. *brachyurus*. This hypothesis is supported by the sympatric distribution of *N. eugenii* and *S. brachyurus* in Western Australia [16]. Studies have shown that most macropodid nematodes undergo speciation *via* host-switching due to overlapping host-ranges and feeding habits [25, 26, 31]. In the present example, apart from infecting a new host species, the parasite appears to have undergone a degree of genetic differentiation within the new host.

In this study, the genetic variation in *A. thylogale* may represent another example of cryptic species as the specimens genetically characterised herein had very similar morphological features [14]. The existence of cryptic species amongst nematodes in macropodid hosts has been detected previously [17, 32, 33]. For example, Chilton et al. [24] reported four sibling species within *Cloacina petrogale* Johnston & Mawson, 1938 using multilocus enzyme electrophoresis. Subsequently, they used DNA sequence data to demonstrate the existence of cryptic species within four morphospecies of the genus *Cloacina* that occurred within rock wallabies of the genus *Petrogale* Gray. More recently, Chilton et al. [26] proposed 10 cryptic species within the phascolostrongyline nematode, *Hypodontus macropi* Mönnig, 1929 from 12 macropodid hosts. Although the reasons for the apparent common occurrence of sibling species in cloacinine and phascolostrongyline nematodes are not clear, the relatively rapid radiation of the hosts [34], as well as the opportunities for host switching, may contribute to this phenomenon [35]. However, the confirmation of these inferences requires further studies with larger sample size.

## Conclusions

A molecular-phylogenetic approach using ITS sequences revealed genetic variation in 37 specimens of *A. thylogale* from *N. eugenii* and *S. brachyurus* from different locations. Phylogenetic analyses of the sequence data revealed distinct clades based on hosts. Additional morphological and molecular studies are required to fully confirm the presence of a new species or cryptic species of *Austrostrongylus*.

## Supplementary information

**Additional file 1: Figure S1.** Alignments of the first (a) and second (b) internal transcribed spacers of *Austrostrongylus thylogale*. A dot indicates an identical nucleotide with respect to the top sequence for each alignment. IUPAC codes indicate polymorphic positions in the sequences.

## Data Availability

All data generated or analysed during this study are included in this published article. DNA sequence data generated during this study are available from the GenBank under the accession numbers MT022443-MT022454.

## Abbreviations

ITS1: first internal transcribed spacer
ITS2: second internal transcribed spacer
PCR: polymerase chain reaction
BI: Bayesian Inference
NJ: neighbour-joining
IUPAC: International Union of Pure and Applied Chemistry
