# Supplementary material for: Genetic variation in Austrostrongylus thylogale Johnston & Mawson, 1940 (Nematoda: Trichostrongylida) from the tammar wallaby, Notamacropus eugenii (Gray), and the quokka, Setonix brachyurus (Quoy & Gaimard) (Marsupialia: Macropodidae) in Australia
Source: Parasit Vectors. 2020 Mar 14;13:135. doi: 10.1186/s13071-020-4007-5 (PMC7071675; doi:10.1186/s13071-020-4007-5)
Supplement: Supplementary file 1 — Additional file 1: Figure S1. Alignments of the first (a) and second (b) internal transcribed spacers of Austrostrongylus thylogale. A dot indicates an identical nucleotide with respect to the top sequence for each alignment. IUPAC codes indicate polymorphic positions in the sequences. [file 13071_2020_4007_MOESM1_ESM.docx]

**Additional file 1: Figure S1**

**a**

10 20 30 40 50 60 70 80 90 100

....|....|....|....|....|....|....|....|....|....|....|....|....|....|....|....|....|....|....|....|

**18B2.11** **TCGTCGAAACCATTCATGGTTCTGTTGATCAAAACGAGAAACCAACACGCGTGTTCTTTGCGACTTTGTCGTCAAAGGTTGGGAGTATCACCCCCGTTAG**

**18B2.14** **....................................................................................................**

**18B2.18** **....................................................................................................**

**24H5.6**  **.....................................................C..............................................**

**24H5.8**  **.....................................................C..............................................**

**24H5.11** **.....................................................C..............................................**

**24J5.4**  **....................................................................................................**

110 120 130 140 150 160 170 180 190 200

....|....|....|....|....|....|....|....|....|....|....|....|....|....|....|....|....|....|....|....|

**18B2.11** **AGCTCTACGTAAGGTGTCTATGTATGGCATGAGTCGTTCTTGAGTGACGGCTATGATTGTCCATGCGAAGTTCCCATTYATTTGGTTGAGCTTCTTCGAC**

**18B2.14** **....................................................................................................**

**18B2.18** **....................................................................................................**

**24H5.6**  **....................................................................................................**

**24H5.8**  **...........................................S........................................................**

**24H5.11** **....................................................................................................**

**24J5.4**  **....................................................................................................**

210 220 230 240 250 260 270 280 290 300

....|....|....|....|....|....|....|....|....|....|....|....|....|....|....|....|....|....|....|....|

**18B2.11** **TTTATGAGCATTGCTAGAATGCCGCCTTACAGCATGTTTTGTCAATTGGTGATTATGCATTTGCATGAAATGCGATACCTGATTTAATCGGGAAATCTTA**

**18B2.14** **....................................................................................................**

**18B2.18** **....................................................................................................**

**24H5.6**  **.....................................................................................G..............**

**24H5.8**  **...........................................M.............................R...........G..............**

**24H5.11** **.......K.............................................................................G..............**

**24J5.4**  **....................................................................................................**

310 320 330 340 350 360 370 380 390

....|....|....|....|....|....|....|....|....|....|....|....|....|....|....|....|....|....|....|....

**18B2.11** **ATGATCCGTGCTAGTTTAACGGACGCCAATACAAAACTCAAACCAATGTTTCATTTATTGATGTTTGCAGAAACGTGACCTWCTGGTTACAACTATGAA**

**18B2.14** **....................................................W...........................................R..**

**18B2.18** **................................................................................................R..**

**24H5.6**  **.................G.................................................................................**

**24H5.8**  **.................G.................................................................................**

**24H5.11** **.................G.................................................................................**

**24J5.4**  **...................................................................................................**

**b**

10 20 30 40 50 60 70 80 90 100

....|....|....|....|....|....|....|....|....|....|....|....|....|....|....|....|....|....|....|....|

**18B2.11** **AACGTACTACTACAGTGTGGCYTGTAATGCACTGTTTGTCGAATGGCACTCATCGTTTAGATGATTCCCATTTCAGTCAAGAAATCGTAGCAACATGGTG**

**18B2.18** **....................................................................................................**

**18B2.19** **....................................................................................................**

**25J5.4**  **....................................................................................................**

**25H5.2**  **.........................................................C..........................................**

110 120 130 140 150 160 170 180 190 200

....|....|....|....|....|....|....|....|....|....|....|....|....|....|....|....|....|....|....|....|

**18B2.11** **TGTGTGCGTATGCTTGTCGCGTACATACCCCTGAATGATGTGAACGTRTGTTGTTACCGTCTCGAATAGTACTCGGCGAGTATGAGGATGTTCGGATGGG**

**18B2.18** **.................T................................A.................................................**

**18B2.19** **....................................................................................................**

**25J5.4**  **....................................................................................................**

**25H5.2**  **....................................................................................................**

210 220 230 240

....|....|....|....|....|....|....|....|

**18B2.11** **GACATGTATGACAACATGATTGTTCTGGCATCATTTGTAT**

**18B2.18** **........................................**

**18B2.19** **........................................**

**25J5.4**  **........................AC..............**

**25H5.2**  **........................................**

**Additional file 1: Figure S1.** Alignments of the first (**a**) and second (**b**) internal transcribed spacers of *Austrostrongylus thylogale*. A dot indicates an identical nucleotide with respect to the top sequence for each alignment. IUPAC codes indicate polymorphic positions in the s
